# Living and working in rural healthcare during the COVID-19 pandemic: a qualitative study of rural family physicians' lived experiences

**DOI:** 10.1186/s12875-022-01942-1

**Published:** 2022-12-22

**Authors:** Nahid Rahimipour Anaraki, Meghraj Mukhopadhyay, Yordan Karaivanov, Margo Wilson, Shabnam Asghari

**Affiliations:** 1 Centre for Rural Health Studies, Faculty of Medicine, Memorial University of Newfoundland, St. John’s, Newfoundland and Labrador, Canada; 2 Labrador‐Grenfell Health, Memorial University of Newfoundland, Newfoundland, Canada; 3 Discipline of Emergency Medicine, Faculty of Medicine, Memorial University of Newfoundland, St. John’s, Newfoundland and Labrador, Canada; 4 Discipline of Family Medicine, Faculty of Medicine, Memorial University of Newfoundland, Faculty of Medicine Building | Room M5M107, 300 Prince Philip Drive, St. John’s, Newfoundland, A1B 3V6 Canada

**Keywords:** COVID-19, Rural Family Physicians, Community-based participatory research, Virtual care, Shortage of staff

## Abstract

**Background:**

The COVID-19 pandemic has been pervasive in its impact on all aspects of Canadian society. Along with its pervasiveness, the disease provided unprecedented complexity to the Canadian healthcare infrastructure, eliciting varying responses from the afflicted healthcare systems in Canada. However, insights into the various parameters and complexities endured by Canadian rural physicians and rural healthcare institutions during the pandemic have been scarce.

**Objective:**

This paper explores the conditions and complexity of living and working of Rural Family Physicians (RFPs) in rural healthcare in Canada during the pandemic.

**Methods:**

Community-based participatory research was utilized as a collaborative and partnership approach, equitably engaged community members in all aspects of research, ranging from designing the research question to analyzing data. Participants of this study include RFPs with at least one year of experience working in rural Canada. Data were collected through telephone interviews and analyzed according to the six-phase guide for the data's inductive thematic analysis. Data collection halted upon saturation.

**Results:**

Five significant compiled categories reflect the lived experiences of Rural Family Physicians. 1- virtual care as a challenge or forward progress; 2- canceling in-person visits and interrupting the routine; 3- shortage of health care providers and supporting staff; 4-ongoing coping process with the pandemic guidelines; 5-COVID-19 combat fatigue.

**Discussion:**

The inception of COVID-19 has significantly impacted rural physicians across several interconnected issues. This study illuminates the lesser-known effects of the COVID-19 pandemic, which heavily impacts rural healthcare.

## Background

The ongoing worldwide disease known as COVID-19 had widespread impacts on how we live and work, leaving almost no aspects of life untouched. Countries have put many measures in place, especially in healthcare systems, to control the spread of the disease. The immediacy and unprecedented nature of the pandemic influenced several structural changes to healthcare systems in both urban and rural populations. For example, there has been a massive trend of virtualization of healthcare service channels [1–3]. By placing significant pressure on physicians and patients through rapidly implementing new procedures, suspending some services, or virtualizing the mode of delivery, the work-life of physicians and the provision of healthcare services have been dramatically affected.

While nearly 20% of Canadians live in a rural areas, only 9.3% of physicians are employed in rural settings [4]. Chauhan et al. (2010) report that in the following two years, one in seven rural physicians plans to leave practices in rural areas [5]. Ng et al. (1997) indicated that there is less than one physician for 1000 people in rural areas, compared to more than two physicians for 1000 people in urban areas [6]. While a family physician shortage is detrimental to the entire Canadian population, it disproportionately impacts rural and remote areas as they have fewer physicians that provide a wide array of services [7].

Before the pandemic, the pre-existing health complications in the demographics of rural Canada demonstrate a higher degree of intricacy in planning and provisioning healthcare in the respective areas [8]. Since its inception, the pandemic has increased the pre-existing complications in rural and remote communities across Canada [9]. The complexity of the COVID-19 pandemic is undeniable in rural and remote areas. In Canada, RFPs endure a lack of staff and medical resources, remoteness, and higher workloads and burnout rates. The pandemic has exasperated demands on RFPs and created extra pressures [10]. These areas constitute a higher rate of chronic diseases, lower life expectancy, care delivery challenges, and shortages of healthcare resources (e.g., facilities, personnel, and more) [8, 11–14].

Despite the harsh severity of the impact of the COVID-19 pandemic on rural communities and their healthcare system, few research initiatives have occurred to drive insights and explore the workplace of rural physicians. Therefore, we aim to illuminate the impacts of the COVID-19 pandemic on the work and lives of RFPs in rural healthcare.

## Methods^1^

We initiated community-based participatory research (CBPR) study by inviting rural Canadian family physicians (RFPs) to contribute their expertise and be involved in decision-making in all aspects of this study. For the purpose of this study, the definition of rural community from Statistics Canada is used which includes "the population living in towns and municipalities outside the commuting zone of larger urban centres (i.e., outside the commuting zone of centres with population of 10,000 or more)” [15]. Additionally, at the beginning of the interviews we asked the physicians if they identified themselves as a rural family physician. Members of community have an opportunity to have various level of involvement. They had one or both of the following roles in this study: as an interviewee (i.e., eleven RFPs) or as a member of an advisory team (i.e., two RFPs) to engage in developing the interview guide, recruiting participants, and collecting and analyzing data. The research design and question guide were developed through the constant collaboration between researchers and an advisory team. All research team members checked the number of questions and wording of the interview guide via email and virtual meetings. After reaching a final consensus, the number of questions increased from 3 to 10. During these procedures, we ensured a consensus between all research team members (i.e., researchers and the advisory team). The CBPR approach fosters partnerships. Therefore, the approach provides a valuable method for developing insights and a better comprehension of a given phenomenon. Israel et al. (2001.p.182) defined CBPR in public health as focusing on “social, structural, and physical environmental inequities through active involvement of community members, organizational representatives, and researchers in all aspects of the research process. Partners contribute their expertise to enhance understanding of a given phenomenon and integrate the knowledge gained with action to benefit the community involved” [16]

Study participants were RFPs with at least one year of experience working in rural Canada. Physicians with a restricted license to practice, locum physicians, and physicians in rural areas for less than one year were excluded. Recruitment for participants occurred via email. The invitation was sent through the Research Capacity Building Programs (RCBP) at Memorial University of Newfoundland and the Society of Rural Physicians of Canada's RuralMed listserv to RFPs in Canada [17, 18]. The advisory team was actively involved in a recruitment procedure by suggesting different recruitment strategies and sharing the recruitment letter among colleagues who meet the required criteria for this study. Snowball sampling was also utilized, where interviewees contributed to identifying potential participants. The recruitment process continued until the researcher “sees similar instances over and over again.” In other words, no further information is obtained, and saturation is achieved. In this study, we reached saturation after nine interviews; however, two additional interviews were conducted to “look for groups that stretch diversity of data as far as possible, just to make certain that saturation is based on the widest possible range of data on the category” [19]. We conducted a qualitative, semi-structured, 30-min telephone interviews to collect data. The sampling strategies included purposive (e.g., gender and years of practice) and snowball sampling. Potential participants screened and selected to ensure a diverse sample. We followed Braun and Clarke's (2006) six-phase guide for the data's inductive thematic analysis (TA) [20]. The extracted themes and codes were presented and discussed with the researchers and advisory team through virtual meetings and email to reach a consensus. The themes were constructed and developed based on open dialogue, consensus and received feedback from participants of this study (i.e., advisory team and interviewees). Following our analysis, to ensure we appropriately captured physicians’ perspectives the summary of the results was shared with the participants for the purpose of member checking, which resulted in receiving positive feedback from several participants who not only supported the extracted categories but also suggested some complementary information. The procedure also assisted in enhancing the credibility of the findings.

Although the primary goal of this study is to explore and understand participants' viewpoints and provide rich information about the context, the importance of generalizability is undeniable. Generalizability is an inseparable ingredient of qualitative studies to advance “the counselling profession” and “scientific knowledge by extracting, analyzing, and synthesizing findings across several studies for a particular phenomenon within similar settings” [21]. Inferential generalizability will apply to this study [22]. The researcher invites transferability by providing comprehensive contextual information and a description of the phenomenon for the reader “to determine the extent to which findings apply from one situation or context” [21, 23].

## Results

Thirteen RFPs were recruited via email across Canada (east, central, and west). The participants consisted of six males and seven females ranging in age from 35 to 65 years old with 5 to 35 years of experience in rural practice. Five categories have been extracted from data: 1- Virtual care as a challenge or forward progress; 2- Canceling in-person visits and interrupting the routine; 3- Shortage of health care providers and supporting staff; 4-Ongoing coping with the pandemic guidelines; 5-COVID-19 combat fatigue.

### Virtual care as a challenge or forward progress

Rural and remote communities have been struggling with various difficulties associated with accessing healthcare. These difficulties include limited access to public transportation; the time and cost of travelling long geographical distances; out-of-pocket expenses; time away from work, and mobility impairments. One of the most predominant advantages of virtual healthcare during the pandemic has been the ability to provide rural residents with accessible and convenient healthcare services.

*“I think, for many in the rural area…, it is nice to save a whole ton of time travelling and some of them work in a different community than my office might be in, which has been a good thing. I think for me too… because sometimes virtual care is faster… So, instead of having a 3-4 weeks wait time to come in, I can see people within a few days usually when they want an appointment”*

Virtual care has brought some advantages for physicians as well. Working at home and interacting with family members while having more autonomy on working hours were highly appreciated by physicians. However, despite the promises and advantages of virtual health care, the downsides are equally outstanding. Besides making clinical decisions and detecting diagnoses over the phone, physicians are constantly struggling with their patient's health literacy. Vague symptoms such as dizziness or skin rashes and the lack of visual cues (e.g., body language and facial expressions) amplify this difficulty. Some patients cannot describe their symptoms or pronounce their medications over the phone.

*“They are trying to tell me like what medications they are taking, and they cannot pronounce the names of the medications.”*

Although virtual care employs different communication services (e.g., video calls, phone calls, and chatting), phone calls are the most available and accessible, yet with challenges including inadequate technological devices and literacy, unreliable cell-service connectivity, and limited calling plans. Due to poor connectivity or calling plans, physicians have been frustrated by frequently disrupted phone call appointments.

*“I think the other thing is that many people added their Internet or phone connection. Their cell service is just not good where they live. So, we are asking them to do that, but then at the same time, they do not have the resources, or some financially do not have- like, they run out of minutes on their phone or things like that.”*

### Canceling in-person visits and interrupting the routine

Physicians have expressed concerns regarding the consequences of cancelling lab tests such as pap smears and mammograms as part of the regular and routine screening procedures that occur every 6 to 12 months. Also, patients with critical medical conditions (e.g., cancer) have dramatically suffered from decreased hospitalization opportunities, delays, and rescheduling of surgical appointments. Additionally, cancelling in-person visits, a lack of information about virtual care, the fear of being infected in emergency departments, and the lack of guidance on the importance of receiving medical care have been expressed to have irreversible consequences for patients with chronic diseases.

*“At the beginning of the pandemic, there was a perception among patients that it was not safe for them to go to the emergency department… that their family doctors were not seeing patients or did not know where to go. So, they ended up getting much sicker- people with chronic diseases get much sicker before they went to the hospital and a lot more people were admitted to the hospital; it would not have been… I mean- I think if there had been better communication to people with chronic diseases about the importance of getting stuff checked out… that it was better to get it checked out rather than waiting until you were so… so- so sick.”*

Furthermore, due to cancelling in-person visits, patients who intended to have face-to-face visits were suggested to go to the emergency department. However, RFPs in emergency departments did not possess access to the patient’s medical history. Due to the lack of access, patients were not subject to timely care. Therefore, family physicians operating in emergency rooms (ERs) follow up with patients to avoid any consequences of delayed follow-ups. However, this is not part of the clinical goals and objectives of ERs.

*“FP [Family physician] not seeing patients in person - telling them to 'go to the ER' if they wanted the face-to-face care. we do not even know them, their medical history, previous investigations, etc., because ER departments do not have access to their FP charts, etc. No one to follow up with investigations or a follow-up appointment due to reduced accessibility or just no family doctor… delayed follow-up and ER physician access to FP via phone/internet, etc. Not knowing whether the patient will get follow-up at all… Then, who is responsible for the test results, ensuring that the patient is seen expediently, etc.? Places big stress on ER doctors who fear these patients will fall through the cracks. The choice then is only to bring them back to the ER ( overloading it) for follow-up - which is not the purpose of the ER department but seemed the only assurance for follow-up/timely care.”*

### Shortage of health care providers and supporting staff

A shortage of physicians and administrative/support staff during the pandemic established many complaints about the workload of RFPs. The shortage of physicians in rural communities only intensified due to the pandemic. COVID-19 restrictions and guidelines (e.g., travel restrictions and quarantine guidelines) dramatically decreased the number of locum physicians. Also, early retirement and resignations due to heavy workloads, excessive stress, and burnout were among the most common reasons for staff shortages. Additionally, many physicians with particular health issues (e.g., autoimmune conditions) have been on leave during the pandemic.

*“Well, we have got a very fragile system here. So, we do not have enough physicians to start with… very fragile and it has worsened. People, I think, have just become burned out. So, we have had a few in the last month… we have had – somebody is leaving; somebody is retiring; somebody else just retired. So, we lost three physicians in a month. So, that is a huge challenge…”*

As a result of restrictions, many patients receiving long-term care in hospitals did not have caregiver, community or family support systems. The lack of support enhanced the burden on nurses and physicians. Furthermore, devoting increased attention to patients who require long-term care (e.g., dementia) restricts focusing care on acutely ill patients who require constant monitoring.

*“I think for me, in the practice that I was doing because, as I say- a number of the people in our hospital are waiting for long-term care. Number one: visitors being unable to come into the hospital… for patients with dementia, to lose that contact with families was devastating. Two: for the nursing staff to lose those caregivers as support was devastating… and because of the loss of those family members and caregivers. Being able to be with those in the hospital meant that nursing staff time was taken away from acute care patients. There were times when I had a patient who was acutely ill [who] needed more close monitoring and [I] was scrambling to try and get the nursing staff to be able to do the things that I needed. However, they were trying to trace down a patient with dementia who… was trying to leave the hospital or something.”*

Due to the shortage of physicians and administrative staff, RFPs working in clinics were overwhelmed by various clinical and administrative responsibilities (e.g., requesting and completing medical records, sending consult notes, setting up consultant appointments, patient contacts, charting, and answering phones). They not only had to shoulder the burden of excessive responsibilities but also must handle the significant pressure and stress of not infecting their family members.

*“I went into the clinic and there was hardly any support stuff… it kind of like- it all cleared out… like there was nobody there… It was just like the streets were empty. So, my clinic was empty of support staff, so I needed to answer phones and fax papers- You know, send consult notes or set up appointments with consultants; that kind of stuff and it was a little- It was thin on the ground and that surprised me and I was in essence, working by myself. They did not even turn the lights on. The hallways were dark! It was really- it was very lonely actually. I often worked there by myself till 7:00 P.M. because of some of the things the support staff would do. I had to find an alternative pathway to writing letters; making sure that the messages I was sending were followed-up; those kinds of things because I was uncertain about having support...”*

### Ongoing coping process with the pandemic guidelines

Frequently changing healthcare policies and regulations occurred during the early stages of the pandemic. According to the interlocutors, these changes confused physicians and patients who had to adjust frequently.

*“So, most people were screened pretty well, but early on, I mean most people did not know what was happening. There was a lot of confusion about who needed to be tested [and] who did not need to be tested… in a small community- in a small hospital communication, confusion about policies early on was challenging”*

The circumstance was further complicated due to most COVID-19 policies and regulations catering to urban areas. Thus, rural healthcare clinics adjusted the policies or developed more contextualized alternatives.

*“The protected code blue guidelines policy- whatever it is called. It was built or developed for urban centers because I think the minimum number of people it called for was something like 5- where… overnight, there are two staff members in the building and then if they need to call the doctor, the doctor comes in. So, for policy, by default, that minimum required 5 humans when we only have a max of three. It made us very sad on one side that they have not thought about the rural aspect.”*

This situation becomes more complex with physicians and patients who have not adopted pandemic guidelines regarding personal health safety (e.g., wearing masks or getting vaccinated). Physicians face difficulties with patients who are skeptical of news about the pandemic and refuse to get vaccinated regardless of their advice. A study participant shared a story of a patient who refused to get vaccinated. After a couple of days, the patient and her husband, hospitalized and left their worried children home alone. The patient survived but still faces challenges in adjusting to life post-treatment. The daily process of advising and convincing patients to follow health safety guidelines and protocols is challenging. Furthermore, most participants experienced conflict with at least one colleague who resisted adopting the safety guidelines. Usually, these conflicts are resolved after a couple of months.

*“Although, I have to say some of the colleagues I was working with never really shift to virtual care; they saw all their patients face-to-face… continued to do that and I think- almost ignored guidelines because it was outside their sense of how to adjust. They were not that adaptable; they just did not adapt!”*

###  COVID-19 combat fatigue

Reorganizing the clinic, modifying the clinical approach, and performing patient screening upon arrival at the clinics have increased workloads and emphasized adverse effects on the well-being of physicians. Following COVID-19 protocols, such as constantly donning and doffing personal protective equipment (PPE), delayed clinical practices and medical examinations increased work fatigue. The adverse effects maximize, in addition to the broadened professional obligations discussed earlier.

*“There is more workplace fatigue with having to work full days in PPE instead of only putting on PPE on a case by case basis and that is the key… from just having the mask and the gloves and goggles on all day. Being a bit more tired with being cautious and not touching your face and other stuff, but also fatigue… when trying to help a patient but knowing that you have delayed starting certain things because of the COVID-19 protocols. For example, making sure that every person gets into full PPE before we start bagging a patient. Well, in a small rural community when you only have so many people, that takes much time. So, you are delaying starting that procedure on a patient by potentially 5-10 minutes until you have enough to be able to do it.”*

Participants frequently discussed their anxiety and stress due to the high risk of contracting the virus at their workplace and transmitting it to their friends and family members. They often described experiencing loneliness stemming from geographical isolation and not having regular support and contact with their colleagues. Resistance to vaccinating or following safety protocols (wearing masks) among patients and colleagues were sources of significant frustration and distress. As illustrated by a participant, physicians feel “defeated” and “helpless” every day since they increasingly witness terrible circumstances in intensive care units.

*“I have never worked harder than in July and I have never been sadder at work. There is a family right now and it is interesting because they… do not believe in the vaccine; like it is a Unicorn or a leprechaun- like it needs your belief to exist and so, they do not believe in it. The wife came in; we had to put her on a ventilator. She had to be ventilated and shipped to the ICU [Intensive care unit] and you know, we brought in the hospital iPad… so her kids could wish her good luck, but you know- potentially saying goodbye to her kids on the iPad, because the kids could not be in the room. All the kids have got COVID-19. The husband now- he is still in ICU.”*

Physicians often discuss that the pandemic intensified burnout and work fatigue, which led to a loss of clinical empathy. The physical and emotional exhaustion of physicians not only harms patient care but also communication with their colleagues. One of the interlocutors expressed that their communications with colleagues have gone “downhill.” Participants believe they are too burnt out and overwhelmed by their workload to provide empathetic care for patients or interact with colleagues effectively.

*“We just work harder which is a short-term solution that eventually impacts patient care because we get tired and we get less empathetic, less compassionate and over time. I think our patient care deteriorates because we are just not there for the patients. It also deteriorates. After all, our communication with coworkers goes downhill because we are just tired and not as empathetic…”*

## Discussion

This study finds five categories that emerged across the broad range of experiences of RFPs working in rural and remote communities in Canada. These categories provide evidence of the impact of the pandemic on the life and work of RFPs.

This study found that despite the financial and time convenience associated with providing healthcare services via virtual care, technological literacy rates increased the complexities in rural and remote communities. Physicians struggled with patients who did not have access to digital resources (for instance, reliable Internet) or the appropriate digital skills. In comparison to urban areas, adopting VHC in rural areas has been less frequent due to limiting technological networks which facilitate channels to rural communities [1]. A primary cause of negative perceptions of VHC by physicians is the potential of outdated technology causing network disruptions and impacting the quality of care they can provide their patients [24]. According to Anaraki et al. (2022), issues such as patients health literacy, technological devices, and unreliable infrastructure should be resolved before implementing virtual care in rural and remote communities [25]. Given the fact that telephone call was the most pervasive means of communication between doctors and patients in rural communities during the pandemic, observing patients’ behaviors to assess the level of health literacy presents a challenge. Although there are a wide variety of tools and tests to assess the health literacy of patients other than behaviours and characteristics observations, context-sensitive tools which address the complexities of clinical practice in rural communities is necessary. Additionally, providing physicians and members of communities with educational and training opportunities at the early stages of a pandemic to ensure they have the required knowledge and skills about virtual visits would have been an asset.

The study found that rural physicians experienced difficulties managing their workload due to shortages in staff during the pandemic. This continuing trend has been increasing over the recent decades [7, 26, 27]. Furthermore, according to the study participants, this shortage has created additional stressors for physicians, as fewer doctors are required to fulfill a broader range of professional demands. Over the pandemic, the shortage of physicians in rural communities increased. The additional stressors led to several early retirements and resignations due to mental health deterioration. This phenomenon coheres with literature that elaborates on the stress experienced by RFPs versus their urban counterparts [28]. In contrasting to the findings by Ing et al. (2020) and Skinner et al. (2019), rural physicians comprise a more vulnerable demographic to COVID-19 infection [29, 30]. Thus, this establishes the susceptibility experienced by rural physicians and the associated rates of departure (retirements and temporary leaves). The current study found that rural physicians were also subject to less support from administrative staff. The lack of support resulted in increased workloads which enhanced mental health stressors.

Additionally, participants of this study have found difficulties with patients and colleagues who did not adopt the safety guidelines of the pandemic. The physicians reported on conflicts with patients who distrust the information and health policy provided by Health Canada. The interlocutors further expressed the lack of clear guidance and policies addressing the health concerns faced in rural communities [31]. Thus, rural healthcare institutions were left to organize and incorporate contextually relevant health safety protocols. More engagement and involvement of rural and remote family physicians, other primary care providers, and members of communities in the entire stages of forming and issuing pandemic response policies and guidelines to better address isolated communities’ priorities would be required.

The findings affirm that rural physicians face significant adverse effects on their well-being. Literature established high levels of mental exhaustion and burnout in Canadian physicians before the pandemic [32–34]. These trends experienced significant increases over the pandemic [35, 36]. The Ontario Medical Association (2021) found a 34.6% increase in severe burnout symptoms from 2020 to 2021 [37]. Binnie et al. (2021) found that two-thirds of Canadian intensive care healthcare workers experienced elevated anxiety [38]. This experienced anxiety is one of the primary stimulators of mental health complications. This study further illuminates factors that amplify physical and emotional exhaustion faced by RFPs, which include complications associated with virtual care and constant precaution over PPE. Ensuring the availability of resources and services at the earliest stages of a pandemic such as courses on pandemic management strategies and stress management strategies to mitigate the adverse impacts of the pandemic on health care providers is necessary.

The study found that the COVID-19 pandemic has induced a multivariate impact on the work-life and practices of rural physicians. More importantly, the pandemic exposed several underlying issues faced by RFPs. Complications with mental health deterioration among rural physicians drastically increased over the pandemic. Furthermore, the lack of contextually relevant policy guidance for rural communities caused additional complexities in rural healthcare. For instance, virtual care is a significant element shaping the future of healthcare and provides accessible and convenient health services, but it is not a universal solution. Some concerns must be addressed before the permanent implementation of virtual care as a substitute for in-person visits in rural/remote communities. These concerns include unreliable infrastructure, lack of technological equipment, patients health literacy, shortage of health care providers, complications accessing medical history, and supporting policies (training, payment, regulation, standards, etc.).

## Limitations

Our findings reflect the lived experiences of Canadian RFPs. The RFPs were in different geographical contexts and shared relatively common experiences of working and living during the pandemic. Though, some hyperlocal factors (e.g., social/ structural and organizational factors related to each community) may not be captured. Additionally, this study was conducted in 2021, one year after the outbreak began in Canada. Even though participants experienced different pandemic phases, they may still not be exposed to the other stages. As we move to a new standard, future studies may explore a full spectrum of RFPs' experiences during different stages– i.e., epidemic, pandemic, and endemic.

## Conclusions

The COVID-19 pandemic has induced a multivariate impact on the work-life and practices of rural physicians. RFPs during the pandemic face unique challenges with unreliable infrastructure, lack of technological devices, patients health literacy, shortage of staff, inapplicable COVID-19 policies and regulations, geographical isolation, and resistance to health safety protocols. The impact has resulted in a long-lasting and profound mental health deterioration that reinforces the importance of developing resources that enhance and maintain resilience among RFPs [10]. The results of this study provide administrators and healthcare regions with comprehensive information on the impact of the COVID-19 pandemic on the living and working conditions of RFPs. The study provides information to guide rural healthcare with contextually relevant interventions for future pandemics.

## Data Availability

The datasets generated and/or analysed during the current study are not publicly available to protect the confidentiality of participants’ data but are available from the corresponding author upon reasonable request.

## Abbreviations

CBPR: Community-based participatory research
ER: Emergency room
FP: Family Physician
ICU: Intensive care unit
PPE: Personal protective equipment
RCBP: Research Capacity Building Programs
RFP: Rural Family Physician
TA: Thematic analysis

## Footnotes

1 All methods were performed in accordance with the relevant guidelines and regulations.
